# State Evaluation of Self-Powered Wireless Sensors Based on a Fuzzy Comprehensive Evaluation Model

**DOI:** 10.3390/s23229267

**Published:** 2023-11-18

**Authors:** Suqin Xiong, Qiuyang Li, Aichao Yang, Liang Zhu, Peng Li, Kaiwen Xue, Jin Yang

**Affiliations:** 1 China Electric Power Research Institute Co., Ltd., Beijing 100192, China; suqinxionggw@163.com (S.X.); 12qiuy.li@gmail.com (Q.L.); 2 State Grid Jiangxi Electric Power Co., Ltd., Power Supply Service Management Center, Nanchang 330032, China; dkyyac2015@163.com (A.Y.); liangzhu202310@163.com (L.Z.); 3 College of Optoelectronic Engineering, Chongqing University, Chongqing 400044, China; 20210801032@cqu.edu.cn (P.L.); 202208131092@stu.cqu.edu.cn (K.X.)

**Keywords:** status evaluation, self-powered wireless sensors, fuzzy evaluation, fusion weights

## Abstract

The energy harvesters used in self-powered wireless sensing technology, which has the potential to completely solve the power supply problem of the sensing nodes from the source, usually require mechanical movement or operate in harsh environments, resulting in a significant reduction in device lifespan and reliability. Therefore, the influencing factors and failure mechanisms of the operating status of self-powered wireless sensors were analyzed, and an innovative evaluation index system was proposed, which includes 4 primary indexes and 13 secondary indexes, including energy harvesters, energy management circuits, wireless communication units, and sensors. Next, the weights obtained from the subjective analytic hierarchy process (AHP) and objective CRITIC weight method were fused to obtain the weights of each index. A self-powered sensor evaluation scheme (FE-SPS) based on fuzzy comprehensive evaluation was implemented by constructing a fuzzy evaluation model. The advantage of this scheme is that it can determine the current health status of the system based on its output characteristics. Finally, taking vibration energy as an example, the operational status of the self-powered wireless sensors after 200 h of operation was comprehensively evaluated. The experimental results show that the test self-powered wireless sensor had the highest score of “normal”, which is 0.4847, so the evaluation result was “normal”. In this article, a reliability evaluation strategy for self-powered wireless sensor was constructed to ensure the reliable operation of self-powered wireless sensors.

## 1. Introduction

With the penetration of the Internet of Things technology into every aspect of our lives, especially in various fields such as the home [1], manufacturing [2], medical [3], retail [4], and agriculture [5], wireless sensor nodes are also widely used, and the accompanying power supply issues are becoming increasingly prominent. The emergence of self-powered sensors has brought a new approach to this issue beyond battery power supply. Self-powered sensors collect energy from environments such as magnetic fields, electric fields [6], vibrations [7], and solar energy [8], and efficiently utilize energy through energy management circuits [9], effectively solving some power supply problems.

However, due to the characteristics of its mechanical structure and the impact of the operating environment, the reliability of self-powered wireless sensors gradually decreases over time during long-term operation, and can even not function properly. Self-powered sensors typically consist of energy collectors, energy management circuits, communication circuits, and sensors [10]. At present, most environmental micro-energy collectors mainly rely on mechanical motion for energy collection, and they may inevitably suffer mechanical damage or even failure during long-term use [11], leading to a decrease in the reliability of energy collection. Self-powered sensors typically require long-term use in environments with a strong electromagnetic [12], humidity [13], and high temperature [14]. Energy management circuits, due to their electrical characteristics, may suffer certain harm when used in harsh environments for a long time, thereby reducing the energy conversion efficiency of energy management circuits [15] and making the output power unstable [16]. Poor environments may reduce communication performance such as wireless communication distance [17]. For commonly used sensors such as temperature [18], pressure [19], and acceleration [20], environmental factors may also lead to a decrease in measurement accuracy and a change in measurement range. However, there are currently no complete and scientific evaluation indexes and comprehensive evaluation methods for self-powered wireless sensors. Most scholars only evaluate the various parts of self-powered wireless sensors [21,22,23,24,25], unable to make scientific and effective judgments on the operating status of self-powered wireless sensors that have been running for a long time, and thus unable to repair and replace them in a timely manner, resulting in the reliability and integrity of user data.

In response to the above issues, the FE-SPS evaluation method proposed in this article aims to fill the gap in the comprehensive evaluation method for the operational status of self-powered wireless sensors. By conducting reliability research based on actual operational data and expert experience from four aspects, i.e., energy harvesters, energy management circuits, wireless communication units, and temperature sensors, a common, simple, scientific, and complete evaluation system and model are constructed to make the evaluation results more accurate and closer to the actual situation. This model can provide timely supplementary suggestions for operation and maintenance personnel, and has reasonable engineering practicality.

This paper is divided into six parts: the first part is the introduction and the second part summarizes the general idea of the FE-SPS evaluation method. The third part introduces the construction of self-powered wireless sensor index system; the fourth part describes the index weighting method of subjective and objective fusion; the fifth part shows in detail the comprehensive evaluation process and experiment of self-powered wireless sensor based on fuzzy evaluation. The sixth part is the conclusion.

## 2. FE-SPS Evaluation Method

**Figure 1:**
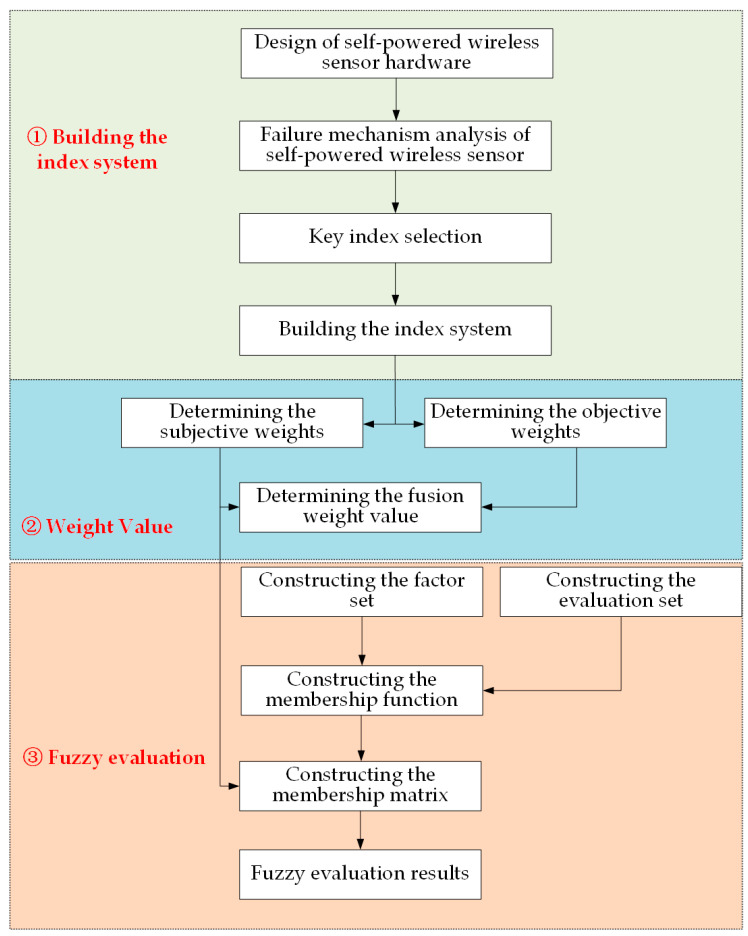
Frame diagram of the FE-SPS evaluation method.

Figure 1 shows the overall block diagram of the FE-SPS evaluation method, which is divided into three steps. Step 1: By analyzing the factors affecting the status and failure mechanism of the self-powered wireless sensor, and selecting the key indexes according to the failure mechanism, the key index system including 4 first-level indexes and 13 second-level indexes was constructed. Step 2: Under the premise of considering subjective and objective weights, the nine-level scale analytic hierarchy process (AHP) and CRITIC weight method were integrated to obtain more comprehensive and scientific evaluation results. Step 3: The factor set and evaluation set were constructed according to the first-level index and the actual experiment scene, and then the corresponding membership degree model was constructed according to the attributes of each index to solve the membership degree matrix, and the membership degree matrix was fused with the fusion weight to obtain the evaluation result.

## 3. Construction of the Failure Mechanism and Index System of the Self-Powered Wireless Sensor

Self-powered wireless sensors can capture weak energy in the environment and convert it into usable energy.

Due to the differences in the environmental energy and circuit methods used, various self-powered sensor systems need to include four parts: energy harvester, energy management circuit, communication mode, and a sensor to achieve the basic self-powered sensing function. Here, a research idea for the state evaluation of self-powered wireless sensors was presented, which could be modified according to the actual equipment used, and the linear vibration energy harvester was taken as an example to carry out related research.

### 3.1. Design of Self-Powered Wireless Sensors

#### 3.1.1. Hardware Design of Self-Powered Wireless Sensors

**Figure 2:**
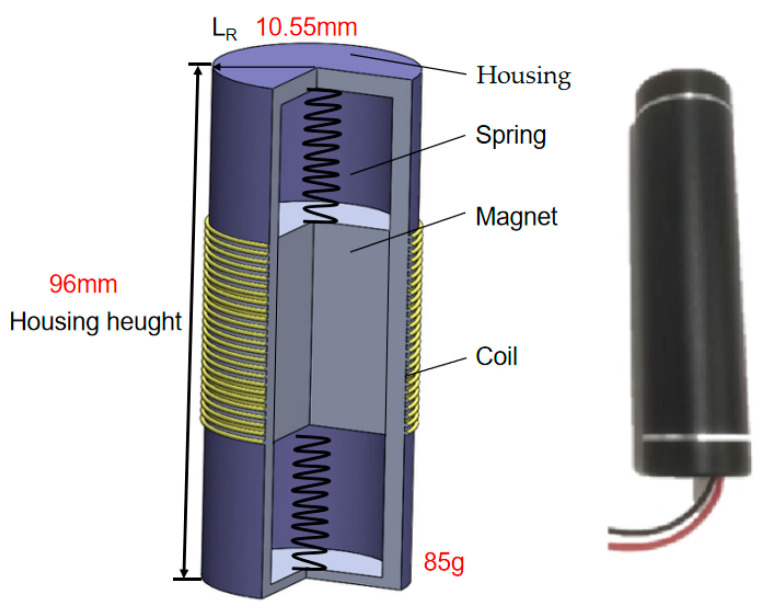
Vibration energy harvester.

The vibration energy harvester used in this article is an electromagnetic linear vibration energy harvester. Compared to piezoelectric and friction nanomotors, the electromagnetic vibration energy harvester has the characteristics of high output power and simple structure, which is easy to process. Figure 2 is the schematic diagram of the vibration energy harvester in this article, which uses a magnet to move back and forth to cut the magnetic induction line and generate energy. Its diameter is 21 mm, height 96 mm, and weight 85 g.

#### 3.1.2. Energy Management Circuit, Wireless Communication Unit, Sensor

**Figure 3:**
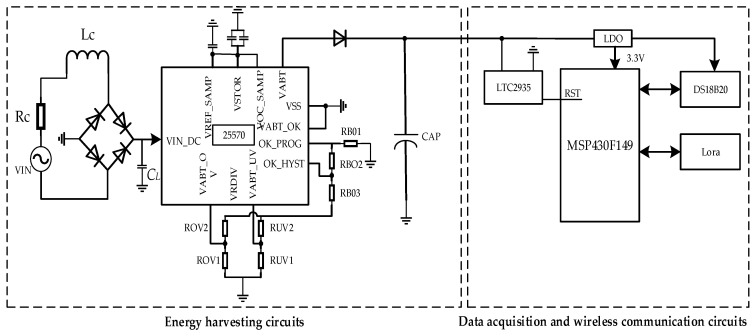
Frame diagram of a self-powered wireless sensor.

Figure 3 shows the design frame diagram and the physical diagram of the self-powered wireless sensor. The test results show that the open-circuit voltage, short-circuit current, and maximum output power of the electromagnetic vibration energy collector were 1.84 V, 12.64 mA, and 23.26 mW, respectively. Therefore, we chose the micro energy chip BQ25570 with a startup voltage as low as 600 mV as the front end of the vibration energy collection circuit to collect and boost the weak vibration energy to 3.6 V. It should be noted that the input of BQ25570 is alternating current (AC) energy, and the AC signal needs to be converted into a direct current (DC) pulsation signal through a rectification circuit. Then, a filtering capacitor CL was used to convert the DC pulsation signal into a DC signal. The capacitor CAP was used to store energy. When the stored energy reached the preset voltage, LTC2935 reset the microprocessor, and then the microprocessor enabled the DS18B20 and Lora module. LTC2935 was used for energy monitoring because the micro-energy in the environment cannot always meet the real-time wireless communication, which requires the energy to be stored and then released to power the load. Here, we used a supercapacitor of 0.33 μF.

#### 3.1.3. Construction of the Testing Platform

**Figure 4:**
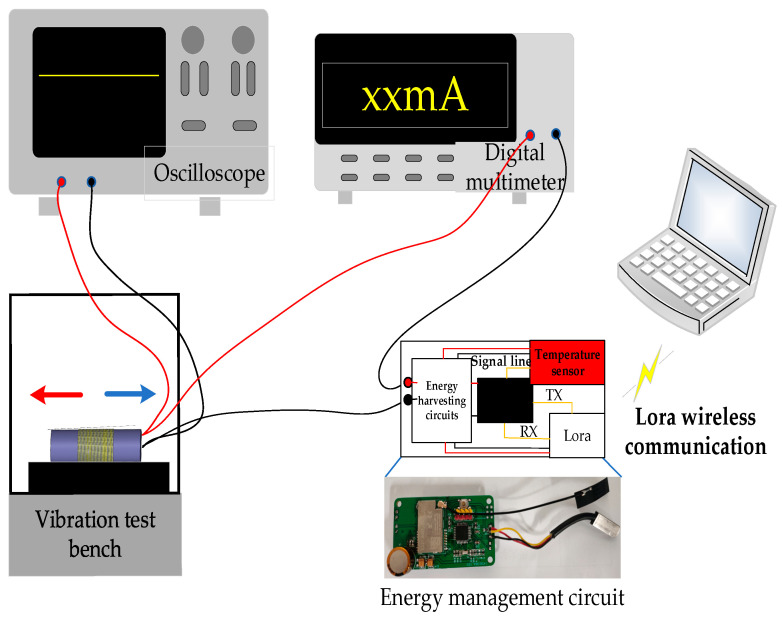
Experimental platform.

In order to evaluate the output capability of self-powered wireless sensors after long-term operation, an experimental platform was built as shown in Figure 4, consisting of a vibration energy harvester, energy management circuit, vibration table, oscilloscope, and digital multimeter. Firstly, the vibration energy harvester was installed on the vibration table, which vibrated back and forth at a speed of 270 mm/s. Then, the digital multimeter was connected to the circuit in a series to measure the output current of the vibration energy harvester, while the oscilloscope was used to measure the voltage value at the moment. The temperature sensor was used to collect the temperature of the indoor environment, and transmits the data to the PC through the Lora wireless communication module for data storage. Among them, the test platform ran for 4 h a day, and a total of 200 h of data needed to be collected. The index parameters of the self-powered wireless sensor were measured every two hours, and the test location was Chongqing University, from June to July 2022.

### 3.2. Failure Mechanism and Key Indexes of the Vibration Energy Harvester 

From Figure 2 and Figure 4, it can be seen that energy is generated by the back-and-forth movement of the internal magnet of the collector under the traction of the spring, cutting the magnetic induction line. This infers that the obstructed movement path of the magnet or changes in the spring’s elastic coefficient will affect the output of the energy harvester, and the repeated mechanical movements will cause wear and tear on the internal structure of the vibration energy harvester. If any connection between the shell, spring, and magnet breaks, it will cause the failure of the vibration energy harvester. 

Due to the complete package of the energy harvester, the mechanical properties of the internal structure of the collector cannot be directly detected, but the health of the energy harvester can be inferred by measuring its electrical properties through the “black box principle”. Among them, changes in the impedance of the spring, shell–spring–magnet connection point, magnet, and coil can cause changes in the open circuit voltage, short circuit current, and equivalent impedance output power of the energy harvester. Based on these four parameters, the following indexes can be obtained:

1. Open-circuit voltage deviation rate

The open-circuit voltage is used to reflect the highest voltage that the energy harvester can output, which is related to the vibration frequency and amplitude. When the vibration frequency and amplitude are the same, the open-circuit voltage of the same batch of energy harvesters should be equal. However, when internal components are damaged or fail, the open circuit voltage will change. If the open circuit voltage is too small, it will not be able to start the energy management circuit, and if it is too large, it may breakdown the energy management circuit. Therefore, the open-circuit voltage deviation rate is introduced here as one of the indexes to evaluate the health status of the energy harvester, which can be expressed by Formula (1):

(1) $$\sigma_{EV}=\left|\frac{V_{0}-V_{t}}{V_{0}}\right|\times 100\%$$

In the formula, $\sigma_{EV}$ is the open-circuit voltage deviation rate, $V_{0}$ refers to the open circuit voltage of the energy harvester at the factory, and $V_{t}$ is the open-circuit voltage of the energy harvester during testing.

2. Short-circuit current deviation rate

The short-circuit current is used to reflect the maximum current that the energy harvester can output, which is affected by the vibration frequency and amplitude, as well as the load carrying capacity. If the short-circuit current is too small, it cannot drive the subsequent load. If internal components are damaged, their output capacity will change. The short-circuit current deviation rate is introduced as one of the indexes to evaluate the health status of the energy harvester, which can be expressed as:

(2) $$\sigma_{EI}=\left|\frac{I_{0}-I_{t}}{I_{0}}\right|\times 100\%$$

where $\sigma_{EI}$ is the short-circuit current deviation rate, $I_{0}$ refers to the short-circuit current of the energy harvester at the factory, and $I_{t}$ is the short-circuit current of the energy harvester during testing.

3. Equivalent impedance deviation rate

**Figure 5:**
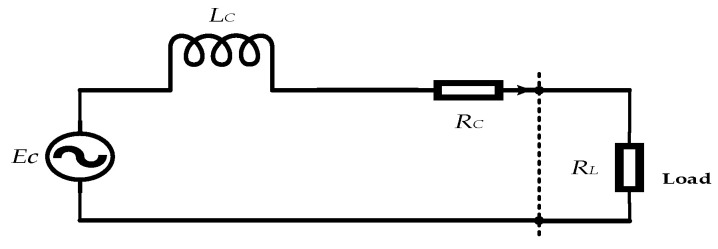
Equivalent circuit diagram of the vibration energy harvester.

When self-powered wireless sensors leave the factory, impedance matching is often performed to ensure that the vibration energy harvester can output at maximum power, where the input impedance of the energy management circuit is equal to the output impedance of the energy harvester. However, when damage occurs inside the energy harvester, the equivalent impedance changes, resulting in a significant decrease in the output capacity of the vibration energy harvester. As shown in Figure 5, the equivalent circuit diagram of the vibration energy harvester is composed of equivalent inductance and equivalent resistance. The vibration energy harvester in this article was mainly used in the application scenario of transmission lines, with a low vibration frequency of 3 Hz–120 Hz. Therefore, the parameter that mainly affects the output performance of the vibration energy harvester is *R_C_*, and *L_C_* can be ignored. Here, the equivalent impedance deviation rate is introduced, which can be expressed by:

(3) $$\sigma_{EB}=\left|\frac{R_{0}-R_{t}}{R_{0}}\right|\times 100\%$$

In the formula, $\sigma_{EB}$ is the bandwidth deviation rate, $R_{0}$ is the resistance of the vibration energy harvester at the factory, and $R_{t}$ is the resistance of the energy harvester during testing.

4. Output power stability

The output power is the product of the output voltage and current of the energy harvester under constant load conditions. Although the open circuit voltage and short circuit current may change when there is some internal damage, if the output power is stable within a certain range, it can still provide stable electricity for subsequent circuits. Therefore, the output power stability is introduced to describe the output energy stability of the energy harvester, which can be expressed as:

(4) $$\varphi_{E}=\left|\frac{P_{\max}-p_{\min}}{\bar{P}}\right|\times 100\%$$

In the formula, $\varphi_{E}$ is the stability of the output power of the energy harvester, $P_{\max}$ is the maximum output power over a period of time, $P_{\min}$ is the minimum output power during this period, and $\bar{P}$ is the average output power during this period.

#### 3.2.1. Ability to Manage Circuit Failure Mechanisms and Key Indexes 

The main function of the energy management circuit is to collect the energy captured by the vibration energy harvester, and boost and store the weak energy. After stabilizing, it provides energy for sensors, microprocessors, and communication modules. Therefore, energy management circuits typically include environmental energy collection units, boost units, and voltage stabilizing circuits. If the self-powered sensor operates in environments such as high temperature, high temperature, high voltage, high electromagnetic, etc., for a long time, it is likely to cause cracks in the circuit packaging, dust layer in the air, water vapor, electromagnetic interference, and other factors can cause changes in the circuit capacitance and resistance, and even cause short circuits and circuit failure. Therefore, the running state of the energy management circuit can be described according to the following three indexes, including output voltage stability, output power stability and energy conversion efficiency.

1. Output voltage stability

The stability of the output voltage of the energy management circuit is a necessary condition to ensure the accuracy of sensors, microprocessors, and wireless communication units, especially for analog sensors that require an analog-to-digital converter (ADC) for data collection. If the reference voltage of the ADC is unstable, it will greatly reduce the accuracy of the sensor. In addition, voltage instability can also lead to the misjudgment of microprocessors and increase the bit error rate and packet loss rate of wireless communication units. Therefore, the output voltage stability is introduced to evaluate the reliability of the energy management circuit, which can be expressed by the following formula:

(5) $$\sigma_{CV}=\left|\frac{\Delta V}{\bar{V}}\right|\times 100\%$$

In the formula, $\sigma_{CV}$ represents the stability of the output voltage over a period of time, ΔV is the range of output voltage of the energy pipeline circuit over a period of time, and $\bar{V}$ is the average value of the output voltage during this period.

2. Circuit output power stability

The power stability of energy management circuits is similar to the definition of the power stability of energy harvesters, both of which are used to describe the degree of dispersion of the output power deviation from the average power value within a certain period of time. The stability of circuit output power is a necessary condition to ensure the normal and stable operation of sensors, microprocessors, and wireless communication units. Therefore, the concept of the output power stability is introduced for the energy management circuit, which can be expressed as:

(6) $$\varphi_{C}=\left|\frac{P_{\max}-p_{\min}}{\bar{P}}\right|\times 100\%$$

where $\varphi_{C}$ is the output power stability of the energy management circuit, $P_{\max}$ is the maximum output power over a period of time, and $P_{\min}$ is the minimum output power during this period.

3. Energy conversion efficiency

Energy conversion efficiency, as one of the most important parameters for various energy collection chips, is naturally one of the key indexes for evaluating self-powered sensors. It can reflect the conversion efficiency of the management circuit in converting input energy into effective energy, and its expression is:

(7) $$\eta_{C}=\frac{P_{\mathrm{out}}}{P_{\mathrm{in}}}\times 100\%$$

In the formula, $\eta_{C}$ is the conversion efficiency of the energy management circuit, $P_{out}$ is the output power of the energy management circuit, and $P_{in}$ is the input power of the energy management circuit.

#### 3.2.2. Key Indexes of the Wireless Communication Unit Failure Mechanism

At present, wireless communication evaluation technology has been widely studied [26,27,28], and a high number of evaluation indexes have been proposed for the evaluation of sensor wireless communication. However, different usage conditions and communication methods have different indexes, so it is not necessary for us to include all indexes. Therefore, indexes need to be selected from the actual engineering background.

The wireless communication unit mainly includes the wireless communication chip and antenna part. Due to the fact that self-powered wireless sensors are usually placed in harsh environments, the antenna is easily damaged, which can lead to the attenuation of the communication distance of the wireless communication unit. At the same time, due to the unstable output voltage and power of the energy management circuit, it can lead to packet loss and bit error in wireless communication data. In addition, different data importance and real-time often require different duty cycles for communication. Therefore, the indexes constructed based on communication distance, packet loss rate, bit error rate, and communication cycle duty ratio are as follows:

1. Communication distance attenuation rate

When the antenna is damaged, its reflection coefficient S11 will be greatly affected, which affects the resonant frequency of the antenna and leads to the attenuation of communication distance. Secondly, some sensors will be placed at the zero point of the communication distance, and once the communication distance of the wireless communication unit decreases, it will directly lead to the failure of the wireless sensor node. At the same time, the electromagnetic interference or the presence of walls in the layout location can also lead to a decrease in communication distance, resulting in information not being transmitted to the user in a timely manner. Therefore, the index of communication distance attenuation rate is introduced to evaluate the health state of the antenna, which can be expressed by the following formula:

(8) $$d_{\eta }=\frac{d_{\max}-d_{t}}{d_{\max}}\times 100\%$$

In the formula, $d_{\eta }$ is the attenuation rate of wireless communication distance, $d_{\max}$ is the maximum communication distance marked by the manufacturer when leaving the factory, and $d_{t}$ is the communication distance at this time.

2. Packet loss rate

The packet loss rate refers to the proportion of the actual number of received data packets to the theoretical number of data packets within a certain period of time. The reason for packet loss is similar to the communication distance. It can be represented by the following formula:

(9) $$DT_{\eta }=\frac{DT_{th}-DT_{t}}{DT_{th}}\times 100\%$$

where $DT_{\eta }$ is the packet loss rate of wireless communication, $DT_{th}$ is the number of theoretically received data packets over a period of time, and $DT_{t}$ is the amount of data actually received over a period of time.

3. Efficiency of data packets

The efficiency and error rate of data packets in this article are similar, referring to the proportion of data packets that are affected by environmental noise, fluctuations in energy management circuit voltage, and other factors during transmission, resulting in signal damage during transmission and generating data that cannot be recognized by users. It can be represented by the following formula:

(10) $$\sigma_{SER}=\frac{DT_{all}-DT_{ser}}{D_{all}}\times 100\%$$

In the formula, $\sigma_{SER}$ is the efficiency of wireless communication data packets, $DT_{all}$ is the number of all data packets over a period of time, and $DT_{ser}$ is the number of data packets that can be recognized by the user over a period of time.

4. Communication timeliness

In general, the transmission power consumption of wireless communication units is high, reaching hundreds of milliamperes. However, the distribution of micro-energy in the environment is random in both time and space, resulting in the inability of the environment energy collected by wireless self-powered sensors to provide real-time power to the wireless communication system. Therefore, supercapacitors/lithium batteries are introduced as energy storage units, and data collection and transmission are carried out when the voltage of the energy storage unit reaches the voltage threshold. If the sending cycle time is too long, it will affect the user’s judgment of the event. Therefore, the communication timeliness index is introduced to evaluate the communication interval time to meet the needs of users. It can be represented by the following equation:

(11) $$\eta_{T}=\left|\frac{\Delta T_{ref}-\Delta T_{t}}{\Delta T_{ref}}\right|\times 100\%$$

where $\eta_{T}$ is the wireless communication timeliness, $\Delta T_{ref}$ is the user specified wireless data transmission interval, and $\Delta T_{t}$ is the actual wireless data transmission interval.

#### 3.2.3. Selection of Sensor Indexes

Sensors can convert perceived physical signals into electrical signals, which is the source of signal acquisition. Therefore, it is necessary to ensure the accuracy of the sensor. Due to the susceptibility of sensors to the measurement environment, especially temperature, humidity, and electromagnetic environment, the sensitive units of the sensors are damaged, resulting in a decrease in the static and dynamic characteristics of the sensors. It is well known that the static characteristics of sensors include parameters such as range, sensitivity, and linearity. However, for users, the most important index is measurement accuracy, which can also reflect parameters such as sensor sensitivity and linearity. Therefore, we chose sensor measurement accuracy as a key index for evaluating the health status of sensors. On the other hand, when the conversion unit of the sensor is damaged, its sensing range will also decrease, so the measurement range is also one of the key parameters for sensor health evaluation.

1. Measurement accuracy

Measurement accuracy, as one of the key indexes of sensor performance, determines the implementation of basic functions. It can be represented by the following formula:

(12) $$\sigma_{s}=\left|\frac{DA_{t}-DA_{\mathrm{ref}}}{DA_{\mathrm{ref}}}\right|\times 100\%$$

In the Formula (12), $\sigma_{s}$ is the measurement accuracy of the sensor, $DA_{t}$ is the actual value measured by users, and $DA_{ref}$ is the value measured by the standard device.

2. Measurement range

The measurement range refers to the difference between the maximum and minimum measurement values that can be measured by the sensor, which can be expressed by the following formula:

(13) $$RA=Q_{\max}-Q_{\min}$$

In the Formula (13), RA is the sensing range of the sensor, $Q_{\max}$ is the maximum value that the sensor can measure, and $Q_{\min}$ is the minimum value that the sensor can perceive. 

**Figure 6:**
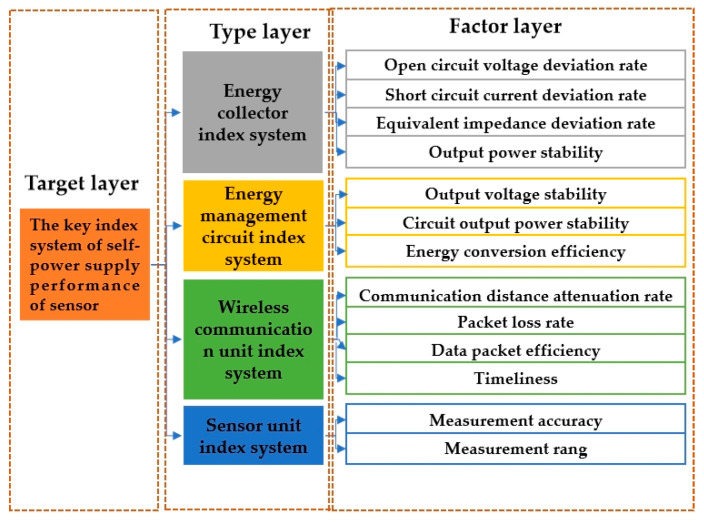
The construction of the index system.

After the above analysis, the self-powered wireless sensor evaluation index system composed of the target layer, type layer, and factor layer was constructed as shown in Figure 6. Among them, the target layer is a Level 1 indicator, which is the evaluation result, used to provide the operational status of self-powered wireless sensors. The type is a Level 2 indicator, which is the type of evaluation, including the energy collector indicator, energy management circuit indicator, wireless communication unit indicator, and sensing unit indicator, used to classify and manage various indicators in the factor layer. The factor layer is a specific evaluation indicator used for the direct evaluation of objectives.

## 4. Index Weighting

Table 1 shows a comparison of classic subjective and objective weighting methods. From Table 1, it can be seen that the analytic hierarchy process (AHP) has the characteristics of simplicity and practicality, and the critical method can consider the variability of indicators while also taking into account the correlation between indicators. Therefore, we chose the AHP as the subjective weight and the critical method as the objective weight in this article. In order to balance the subjective weight of expert experience and the scientific nature of objective weight, a subjective and objective fusion method was adopted in this article to assign weights to various indicators in the evaluation system. We used the fusion weight of AHP and critical algorithm as the weighting method in this article, because the effectiveness and scientific nature of this method have been verified in many fields [29,30]. In the following research, we will further choose some novel weighting methods to obtain more efficient weights, such as the CILOS, IDOCRIW, FUCOM, LBWA, SAPEVO-M, and MEREC algorithms mentioned in the literature [31].

### 4.1. Index Weighting Based on AHP

The AHP is used to determine the subjective weight values of each index. To determine the subjective weight values, it is necessary to first construct a judgment matrix, where experts in this field compare the indexes of each layer in pairs and score the influencing factors of each layer based on the Santy1-9 scale method shown in Table 2 to obtain the judgment matrix of Equation (14) [32].

(14) D=1151216515321511461341

In order to avoid logical errors in the judgment of the supervisor, it is necessary to conduct consistency checks on the obtained judgment matrix, namely:

(15) $$CI=(\lambda_{\max}-n)/(n-1)$$

(16) CR=CI/RI

Among them, $\lambda_{\max}$ is the maximum eigenvalue of the judgment matrix, *n* is the order of the matrix, and *RI* is the average random consistency value corresponding to *n* (Table 3). When the calculated *CR* < 0.1, the consistency test is passed.

Equation (14) is the judgment matrix of each factor in the type layer of the influencing factors. The calculated consistency parameter *CR* = 0.072 < 0.1 indicates that the consistency test has been passed. The other methods for constructing judgment matrices and consistency judgment are consistent with the above methods. After calculation, the consistency parameters *CR* of each factor layer are 0.0442, 0.0904, 0.0986, and 0, respectively.

**Figure 7:**
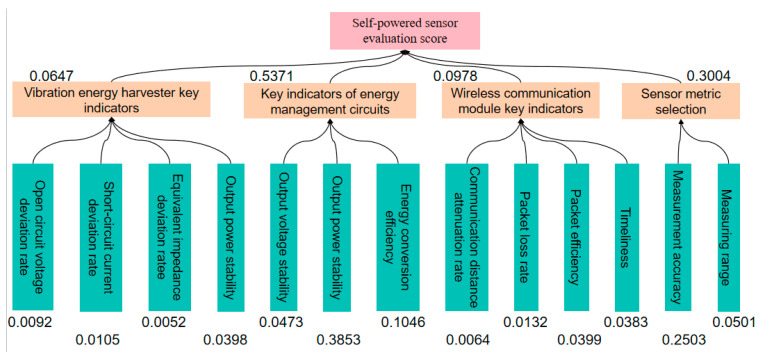
Subjective weight values of indexes at various levels.

After calculation, all the judgment matrices have passed the consistency test, and then the specific weights of each index need to be determined using the root square method.

(17) $${\bar{\omega }}_{i}=\sqrt[{m}]{\prod_{j=1}^{m}a_{ij}}$$

(18) $$\omega_{i}=\frac{{\bar{\omega }}_{i}}{\sum_{j=1}^{m}{\bar{\omega }}_{j}}$$

where *m* is the order of the judgment matrix, $a_{ij}$ is the scale of the elements in the judgment matrix, $\omega_{i}$ is the subjective weight of the i-th factor after normalization, and $\bar{\omega }$ is the subjective weight of the i-th factor before normalization. The subjective weight values of various influencing factors obtained are shown in Figure 7.

### 4.2. Index Weighting Based on the CRITIC Weight Method

The objective weighting method used in this article is the CRITIC weight method, which takes into account both the variability of indicators and their interrelationships [27,28]. The computational steps are as follows:

1. Normalize and standardize each indicator as shown in Equations (19) and (20):

(19) $$x_{ij}^{\prime }=\frac{X_{ij}-\min(X_{1j},X_{2j},\cdot \cdot \cdot ,X_{nj})}{\max(X_{1j},X_{2j},\cdot \cdot \cdot ,X_{nj})-\min(X_{1j},X_{2j},\cdot \cdot \cdot ,X_{nj})}$$

(20) $$x_{ij}^{\prime }=\frac{\max(X_{1j},X_{2j},\cdot \cdot \cdot ,X_{nj})-X_{ij}}{\max(X_{1j},X_{2j},\cdot \cdot \cdot ,X_{nj})-\min(X_{1j},X_{2j},\cdot \cdot \cdot ,X_{nj})}$$

Equations (19) and (20) represent the normalization and standardization processes for positive indicators and negative indicators, respectively. In these equations, $X_{ij}$ represents the *j*-th specific parameter of the *i*-th influencing factor in the factor layer, while $x_{ij}^{\prime }$ represents the parameter after normalization and standardization.

2. Calculate the comparative strength between various indicators, with the calculation process described as Equation (21):

(21) $$\left\{\begin{array}{c} {\bar{x}}_{j}=\frac{1}{n}\sum_{i=1}^{n}x_{ij}\\ S_{j}=\sqrt{\frac{{\sum_{i=1}^{n}\left(x_{ij}-{\bar{x}}_{j}\right)}^{2}}{n-1}} \end{array}\right.$$

In the equation, ${\bar{x}}_{j}$ represents the mean value of each indicator, while $S_{j}$ represents the comparative strength of the indicators.

3. Calculate the conflict of the indicators, with the calculation process described as Equation (22):

(22) $$R_{j}=\sum_{i=1}^{p}(1-r_{ij})$$

In the equation, $R_{j}$ represents the conflict of indicator *j*, and $r_{ij}$ represents the correlation coefficient between the *i*-th and *j*-th factors.

4. Calculate the total amount of information contained in a single indicator, with the calculation process described as Equation (23):

(23) $$C_{j}=S_{j}\times R_{j}$$

In the Equation (23), $C_{j}$ represents the total amount of information contained in a single indicator.

5. Normalize the total amount of information $C_{j}$ and obtain the objective weight values of each factor, with the calculation process described as Equation (24):
  (24) $$w_{j}=\frac{C_{j}}{\sum_{j=1}^{p}C_{j}}$$

The objective weights obtained from 200 h of actual data testing are shown in Table 4.

### 4.3. Index Weighting for Subjective and Objective Integration 

After obtaining the subjective and objective weight values of each factor in the evaluation system, in order to balance the subjective opinions of experts and the objective facts of the data, it is necessary to construct a fusion weight value ($W_{F}$) that can reflect the weight information of subjective weight value ($W_{Z}$) and objective weight value ($W_{K}$). When the sum of distances from $W_{F}$ to $W_{Z}$ and $W_{K}$ is the smallest, it can be considered that the fusion weight value reflects the information of subjective and objective weight values to the greatest extent. The specific calculation process is as follows:

(25) dxy=∑i=1nxi−yip1p,p≥1

The initial fusion weight value is calculated using Equation (25), and then normalized to the formula as in Equation (26).

(26) $$\min D=\sum_{i=1}^{n}\left(\left|W_{Fi}-W_{Zi}\right|+\left|W_{Fi}-W_{Ki}\right|\right)$$

where $W_{Fi}$ represents the fusion weight value after normalization, and $W_{F0j}$ represents the initial weight value.

(27) $$W_{Fi}=\frac{W_{F0j}}{\sum_{j=1}^{n}W_{F0j}}$$

The weight values of each index after subjective and objective fusion are shown in Table 5.

## 5. Fuzzy Comprehensive Evaluation

**Figure 8:**
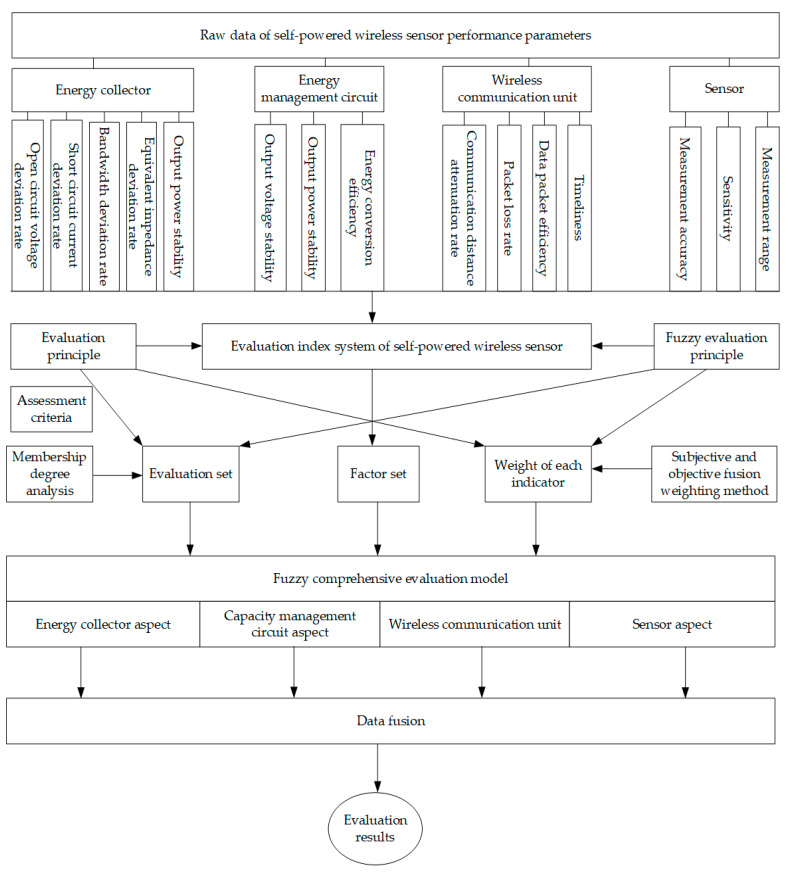
Flow chart of the fuzzy comprehensive evaluation.

Self-powered wireless sensors have numerous operating status parameters, and the operating mechanisms of each part are affected by various factors. Among them, factors related to device health status are mostly characterized by ambiguity and uncertainty, especially for self-powered wireless sensors, there is currently no mature and available index system. Therefore, a fuzzy evaluation model was constructed as shown in Figure 8 to evaluate the operational status of self-powered wireless sensors. The main idea is that a fuzzy comprehensive evaluation is an evaluation method based on the fuzzy mathematics theory. This evaluation method borrows the concept of membership function from fuzzy mathematics, fuzzifying the traditional evaluation method of indexes with fixed values as boundaries, and constructing a membership matrix through the characterization of the membership function. The weight values of each factor are multiplied by the membership matrix to obtain the overall membership evaluation [33]. At the same time, a combination of subjective and objective methods was used to determine the weight values of each factor in order to balance the authority and objectivity of the determined weights. 

It is evaluated by measuring the output parameters of each index of the sensor. The evaluation process includes the hierarchical division of influencing factors, determination of subjective weight values of influencing factors, determination of objective weight values of influencing factors, determination of comprehensive weight values of influencing factors, determination of evaluation membership functions of each influencing factor, and giving comprehensive evaluation scores to self-powered sensors. The flowchart of the evaluation model proposed in this article is shown in Figure 8. 

The steps for fuzzy comprehensive evaluation are as follows:

1. Collection of raw parameters for evaluation indexes

The original data of each index at the 200th hour were taken as the data samples to evaluate the status of the self-powered wireless sensor. The specific data are shown in Table 6.

2. Determine the set of factors

As can be seen from the above parts, the vibration energy harvester, energy management circuit, wireless communication unit, and sensor indexes can all evaluate the operating status of self-powered wireless sensors. Therefore, the factor set can be expressed as:

(28) $$U=\left\{U_{1},U_{2},U_{3},U_{4}\right\}$$

The sub evaluation factors, $U_{i}$, in the factor set can be expressed as:

(29) $$U_{i}=\left\{U_{i1},U_{i2},\cdots ,U_{ij}\right\}$$

where $U_{i}$ is the *i*-th evaluation factor among the main factors, and $U_{ij}$ is the *j*-th evaluation factor in the $U_{i}$ factor set.

3. Determine the comment set

On the basis of summarizing the actual operating experience of self-powered wireless sensors, their operating states are specifically divided into four states: normal, attention, abnormal, and severe. Among them, “normal” indicates that the equipment is working normally and does not require maintenance or attention; “attention” indicates that the equipment is working normally, but requires regular attention and maintenance; “abnormal” indicates that although the equipment is currently functioning normally, but there is a significant risk and needs to be repaired or replaced as soon as possible; “severe” indicates that the device is no longer functioning properly and requires immediate repair and replacement. The comment set is represented as:

(30) $$V=\left\{V_{1},V_{2},V_{3},V_{4}\right\}=\left\{\mathrm{normal},\mathrm{attention},\mathrm{abnormal},\mathrm{severe}\right\}$$

4. Determine the standard values

In order to quantify the status of self-powered wireless sensors, there should be corresponding standard values for each evaluation factor, $U_{ij}$, in various states of the evaluation set. This standard value can serve as an important boundary for distinguishing between good and bad indexes in the program. However, there is currently little research on the evaluation of self-powered wireless sensors, so there is no evaluation standard for self-powered wireless sensors, and there is also no standard value for the status of each evaluation index. Therefore, according to the regulations of the China National Grid for wireless sensors and self-powered sensors, the chip manual, the factory experiment data of each part, and the experts of the National Grid and Chongqing University, the standard values of self-powered wireless sensors based on vibration energy were formulated, as shown in Table 7.

5. Construction of the membership matrix

**Figure 9:**
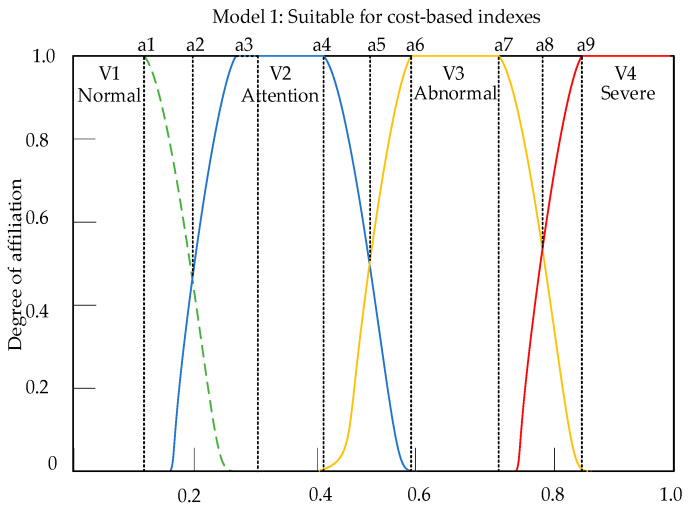
Model 1: Smaller is a better-type function curve.

**Figure 10:**
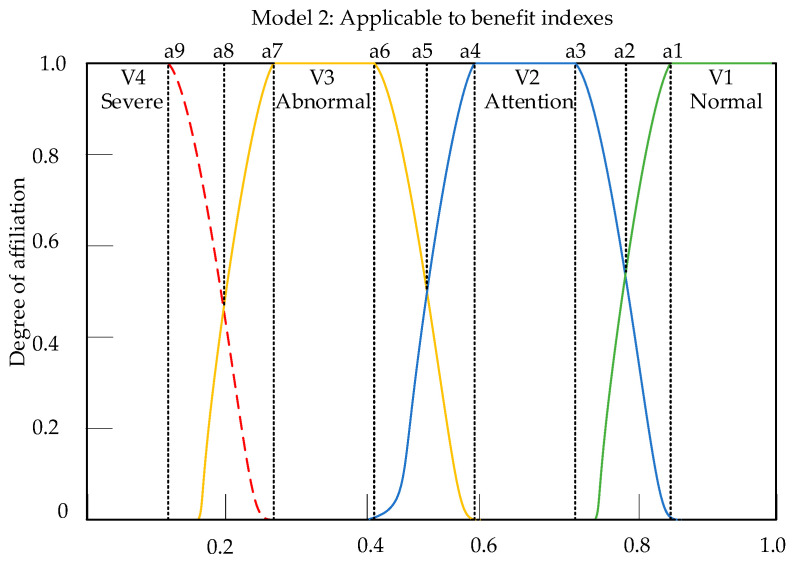
Model 2: Bigger is a better-type function curve.

The membership matrix is a matrix used to determine the degree of superiority or inferiority of specific indexes. Through this matrix, the degree to which a specific index belongs to a certain evaluation level can be understood. Before constructing the membership matrix, it is necessary to construct the membership function. Taking the evaluation object of this article as an example, when the parameters of some indexes fall at the intersection of the two standard values in Table 7, simply dividing them according to intervals will cause the evaluation results to be too extreme, which is not conducive to the comprehensive evaluation of the entire device. Therefore, based on the type of each index, its own membership function for each index was constructed, which can fully play the role of the index throughout the entire evaluation process. Based on the distribution pattern and attributes of the self-powered wireless sensor indexes in this article, the ridge distribution function model as shown in Figure 9 and Figure 10 was selected as the membership function. The indexes can be divided into two categories: cost-based indexes and benefit-based indexes, and their respective membership functions are as follows:

(31) $$\mu_{1}(x)=\left\{\begin{array}{l} \;\;\;\;\;\;\;\;\;\;\;\;1\;\;\;\;\;\;\;\;\;\;\;\;\;\;\;\;\;\;\;\;\;\;\;\;\;\;\;\;\;\;\;\;x\leq a_{1}\\ \frac{1}{2}-\frac{1}{2}\sin\frac{\pi }{a_{1}}(x-a2)\;\;\;\;\;\;\;\;\;\;\;\;\;\;\;a_{1}<x\leq a_{3}\\ \;\;\;\;\;\;\;\;\;\;\;\;0\;\;\;\;\;\;\;\;\;\;\;\;\;\;\;\;\;\;\;\;\;\;\;\;\;\;\;\;\;\;\;\;x>a_{3} \end{array}\right.$$

(32) $$\mu_{2}(x)=\left\{\begin{array}{l} \;\;\;\;\;\;\;\;\;\;\;\;0\;\;\;\;\;\;\;\;\;\;\;\;\;\;\;\;\;\;\;\;\;\;\;\;\;\;\;\;\;\;\;\;x\leq a_{1}\\ \frac{1}{2}+\frac{1}{2}\sin\frac{\pi }{0.2}(x-a_{2})\;\;\;\;\;\;\;\;\;\;\;\;\;\;a_{1}<x<a_{3}\\ \;\;\;\;\;\;\;\;\;\;\;\;1\;\;\;\;\;\;\;\;\;\;\;\;\;\;\;\;\;\;\;\;\;\;\;\;\;\;\;\;\;\;\;\;a_{3}<x\leq a_{4}\\ \frac{1}{2}-\frac{1}{2}\sin\frac{\pi }{0.2}(x-a_{5})\;\;\;\;\;\;\;\;\;\;\;\;\;\;a_{4}<x<a_{6}\\ \;\;\;\;\;\;\;\;\;\;\;\;0\;\;\;\;\;\;\;\;\;\;\;\;\;\;\;\;\;\;\;\;\;\;\;\;\;\;\;\;\;\;\;\;x\geq a_{6} \end{array}\right.$$

(33) $$\mu_{3}(x)=\left\{\begin{array}{l} \;\;\;\;\;\;\;\;\;\;\;\;0\;\;\;\;\;\;\;\;\;\;\;\;\;\;\;\;\;\;\;\;\;\;\;\;\;\;\;\;\;\;\;\;x\leq a_{4}\\ \frac{1}{2}+\frac{1}{2}\sin\frac{\pi }{0.2}(x-a_{5})\;\;\;\;\;\;\;\;\;\;\;\;\;\;a_{4}<x<a_{6}\\ \;\;\;\;\;\;\;\;\;\;\;\;1\;\;\;\;\;\;\;\;\;\;\;\;\;\;\;\;\;\;\;\;\;\;\;\;\;\;\;\;\;\;\;\;a_{6}<x\leq a_{7}\\ \frac{1}{2}-\frac{1}{2}\sin\frac{\pi }{0.2}(x-a_{8})\;\;\;\;\;\;\;\;\;\;\;\;\;\;a_{7}<x<a_{9}\\ \;\;\;\;\;\;\;\;\;\;\;\;0\;\;\;\;\;\;\;\;\;\;\;\;\;\;\;\;\;\;\;\;\;\;\;\;\;\;\;\;\;\;\;\;x\geq a_{9} \end{array}\right.$$

(34) $$\mu_{4}(x)=\left\{\begin{array}{l} \;\;\;\;\;\;\;\;\;\;\;\;0\;\;\;\;\;\;\;\;\;\;\;\;\;\;\;\;\;\;\;\;\;\;\;\;\;\;\;\;\;\;\;\;x\leq a_{7}\\ \frac{1}{2}+\frac{1}{2}\sin\frac{\pi }{a_{1}}(x-a_{8})\;\;\;\;\;\;\;\;\;\;\;\;\;\;\;a_{7}<x\leq a_{9}\\ \;\;\;\;\;\;\;\;\;\;\;\;1\;\;\;\;\;\;\;\;\;\;\;\;\;\;\;\;\;\;\;\;\;\;\;\;\;\;\;\;\;\;\;\;x>a_{9} \end{array}\right.$$

(35) $$\mu_{1}(x)=\left\{\begin{array}{l} \;\;\;\;\;\;\;\;\;\;\;\;0\;\;\;\;\;\;\;\;\;\;\;\;\;\;\;\;\;\;\;\;\;\;\;\;\;\;\;\;\;\;\;\;x\leq a_{3}\\ \frac{1}{2}+\frac{1}{2}\sin\frac{\pi }{a_{1}}(x-a_{8})\;\;\;\;\;\;\;\;\;\;\;\;\;\;\;a_{3}<x\leq a_{1}\\ \;\;\;\;\;\;\;\;\;\;\;\;1\;\;\;\;\;\;\;\;\;\;\;\;\;\;\;\;\;\;\;\;\;\;\;\;\;\;\;\;\;\;\;\;x>a_{1} \end{array}\right.$$

(36) $$\mu_{2}(x)=\left\{\begin{array}{l} \;\;\;\;\;\;\;\;\;\;\;\;0\;\;\;\;\;\;\;\;\;\;\;\;\;\;\;\;\;\;\;\;\;\;\;\;\;\;\;\;\;\;\;\;x\leq a_{6}\\ \frac{1}{2}+\frac{1}{2}\sin\frac{\pi }{0.2}(x-a_{5})\;\;\;\;\;\;\;\;\;\;\;\;\;\;a_{6}<x<a_{4}\\ \;\;\;\;\;\;\;\;\;\;\;\;1\;\;\;\;\;\;\;\;\;\;\;\;\;\;\;\;\;\;\;\;\;\;\;\;\;\;\;\;\;\;\;\;a_{4}<x\leq a_{3}\\ \frac{1}{2}-\frac{1}{2}\sin\frac{\pi }{0.2}(x-a_{2})\;\;\;\;\;\;\;\;\;\;\;\;\;\;a_{3}<x<a_{1}\\ \;\;\;\;\;\;\;\;\;\;\;\;0\;\;\;\;\;\;\;\;\;\;\;\;\;\;\;\;\;\;\;\;\;\;\;\;\;\;\;\;\;\;\;\;x\geq a_{1} \end{array}\right.$$

(37) $$\mu_{3}(x)=\left\{\begin{array}{l} \;\;\;\;\;\;\;\;\;\;\;\;0\;\;\;\;\;\;\;\;\;\;\;\;\;\;\;\;\;\;\;\;\;\;\;\;\;\;\;\;\;\;\;\;x\leq a_{9}\\ \frac{1}{2}+\frac{1}{2}\sin\frac{\pi }{0.2}(x-a_{8})\;\;\;\;\;\;\;\;\;\;\;\;\;\;a_{9}<x<a_{7}\\ \;\;\;\;\;\;\;\;\;\;\;\;1\;\;\;\;\;\;\;\;\;\;\;\;\;\;\;\;\;\;\;\;\;\;\;\;\;\;\;\;\;\;\;\;a_{7}<x\leq a_{6}\\ \frac{1}{2}-\frac{1}{2}\sin\frac{\pi }{0.2}(x-a_{5})\;\;\;\;\;\;\;\;\;\;\;\;\;\;a_{6}<x<a_{4}\\ \;\;\;\;\;\;\;\;\;\;\;\;0\;\;\;\;\;\;\;\;\;\;\;\;\;\;\;\;\;\;\;\;\;\;\;\;\;\;\;\;\;\;\;\;x\geq a_{4} \end{array}\right.$$

(38) $$\mu_{4}(x)=\left\{\begin{array}{l} \;\;\;\;\;\;\;\;\;\;\;\;1\;\;\;\;\;\;\;\;\;\;\;\;\;\;\;\;\;\;\;\;\;\;\;\;\;\;\;\;\;\;\;\;x\leq a_{9}\\ \frac{1}{2}-\frac{1}{2}\sin\frac{\pi }{a_{1}}(x-a_{8})\;\;\;\;\;\;\;\;\;\;\;\;\;\;\;a_{9}<x\leq a_{7}\\ \;\;\;\;\;\;\;\;\;\;\;\;0\;\;\;\;\;\;\;\;\;\;\;\;\;\;\;\;\;\;\;\;\;\;\;\;\;\;\;\;\;\;\;\;x>a_{9} \end{array}\right.$$

In the Formulas (35)–(38), x represents the true values of each index parameter, and $a_{1}\sim a_{9}$ are the boundary values of the parameter, indicating which comment belongs to the boundary. 

Next, it is necessary to classify the membership models of each index. The results of the membership models determined for each index in this article are shown in Table 8. The model classification in Table 8 was developed by experts from Chongqing University and then determined after consultation with experts from the State Grid of China.

Finally, in order to construct the membership matrix, the membership matrix is represented by *R*, and is constructed from the factor set U and the comment set V. Each factor in the matrix is determined by the membership function. At the same time, to ensure the consistency of the values in the membership matrix, it is necessary to normalize each row of the matrix. Each row of the membership matrix reflects the membership relationship of an evaluation factor to each review set, $r_{ij}$ is the membership relationship of the *i* factor $u_{i}$ to the *j* comment $v_{i}$, and the specific value is determined by the membership function. The membership matrix *R* is as follows:

(39) $$R=\left[\begin{array}{c} r_{11}\\ \vdots \\ r_{n1} \end{array}\begin{array}{c} \cdots \\ \ddots \\ \cdots \end{array}\begin{array}{c} r_{1m}\\ \vdots \\ r_{nm} \end{array}\right]=\left(\begin{array}{cccc} \begin{array}{c} 0\\ 0.92\\ 1\\ 1\\ 1\\ 1\\ 0\\ 1\\ 0.71\\ 0\\ 0\\ 0\\ 0 \end{array} & \begin{array}{c} 1\\ 0.08\\ 0\\ 0\\ 0\\ 0\\ 1\\ 0\\ 0.34\\ 1\\ 0\\ 0.03\\ 1 \end{array} & \begin{array}{c} 0\\ 0.2\\ 0\\ 0\\ 0\\ 0\\ 0\\ 0\\ 0\\ 0\\ 1\\ 0.97\\ 0 \end{array} & \begin{array}{c} 0\\ 0\\ 0\\ 0\\ 0\\ 0\\ 0\\ 0\\ 0\\ 0\\ 0\\ 0\\ 0 \end{array} \end{array}\right)$$

6. Calculate the evaluation results

After obtaining the subjective and objective fusion weight matrix, *W*, and membership matrix, *R*, in Table 5, the final evaluation vector *B* can be obtained by multiplying them:

(40) $$\begin{array}{l} B=\left(w_{1},w_{2},\cdots ,w_{n}\right)\left[\begin{array}{c} r_{11}\\ \vdots \\ r_{n1} \end{array}\begin{array}{c} \cdots \\ \ddots \\ \cdots \end{array}\begin{array}{c} r_{1m}\\ \vdots \\ r_{nm} \end{array}\right]=\left(b_{1},b_{2},\cdots ,b_{n}\right)\\ =\left(\begin{array}{cccc} 0.4847 & 0.2681 & 0.2476 & 0 \end{array}\right) \end{array}$$

From the evaluation vector *B*, it can be seen that the membership level belonging to the “normal” level is the highest, with a comprehensive score of 0.4847. According to the principle of maximum membership, the final evaluation result is “normal”. This evaluation result is reasonable because the main factors causing equipment degradation are vibration and external environmental factors, and the testing environment in this article is a relatively ideal environment. On the other hand, from the test data, it can be seen that the main factor affecting the performance degradation of self-powered wireless sensors is the vibration energy harvester. After expert analysis, it is believed that the evaluation results are in line with the actual situation, so our evaluation method can scientifically reflect the operating status of self-powered wireless sensors.

## 6. Conclusions

In this work, the failure mechanism and degradation factors of self-powered sensors were analyzed, and it is concluded that the main factors causing equipment degradation are the mechanical structure movement of the equipment and the operating environment. A relatively scientific and complete evaluation index system has been comprehensively considered and constructed from four aspects: vibration energy harvesters, energy management circuits, wireless communication units, and sensors, including 4 types of primary indexes and 13 secondary indexes. A self-powered wireless sensor was designed using a vibration energy harvester as an example, and a vibration measurement platform was built. Long-term testing was conducted in a laboratory environment for 200 h.

The subjective weight value of each index was obtained using the Santy1-9 scale method, and the objective weight of each index was obtained using the critic weight method according to the 200 h of measured data. According to the principle of minimum distance, the sum of the Manhattan distances between the subjective and objective weight values and the fused weight values were used as the fitness function, and further normalization was carried out to obtain the fused weight values, preparing for the subsequent fuzzy comprehensive evaluation.

A factor set and evaluation set suitable for self-powered wireless bed devices were constructed using the fuzzy mathematics theory. The membership matrix was obtained by selecting and adapting ridge shaped membership functions based on the characteristics of each index. Finally, the subjective and objective weights were fused with the membership matrix to obtain the evaluation matrix, and according to the principle of maximum membership, the evaluation result of the self-powered wireless sensor after 200 h of operation was determined to be “normal”.

## Figures and Tables

**Table 1:** Comparison of common subjective and objective weighting methods.

Weight Assignment Method	Subjective/ Objective	Advantage	Disadvantage
Expert scoring method	Subjective	Simple and fast	Highly influenced by expert experience
AHP	Subjective	Simple and practical, systematic, less quantitative data information required	Highly influenced by expert experience, more complex eigenvalue method
Entropy weight method	Objective	Ability to consider uncertainty and information, does not rely on expert judgment	High vulnerability to data errors, correlations between indicators cannot be considered
CRITIC method	Objective	It can simultaneously take into account the variability of indicators and the correlation between indicators, multiple attributes and decision scenarios can be handled	Need a lot of comparative data, professional software support may be required
Variation coefficient method	Objective	Simple and easy to implement, each indicator can be effectively distinguished	There are certain restrictions on the selection of indicators

**Table 2:** Santy1-9 scale method.

Scale	Meaning
1	It means that two elements are of equal importance compared to each other
3	It means that the former is slightly more important than the latter
5	It means that the former is significantly more important than the latter
7	It means that the former is more important than the latter
9	It means that the former is more important than the latter
2, 4, 6, 8	It means the middle value of the above neighboring judgments
The reciprocal of 1 to 9	It means the importance of the exchange order of two factors

**Table 3:** Average random consistency RI values.

Order (*n*)	1	2	3	4	5	6	7
*RI*	0	0	0.52	0.89	1.12	1.26	1.36

**Table 4:** CRITIC weight calculation results.

Item	Index Variability	Index Conflict	Amount of Information	Weight (%)
Open-circuit voltage deviation rate	1.703	12.093	20.594	10.43
Short-circuit current deviation rate	2.535	12.890	32.681	16.56
Equivalent impedance deviation rate	0.614	12.257	7.522	3.81
Output power stability	1.491	12.444	18.559	9.40
Output voltage stability	1.684	12.083	20.346	10.31
Circuit output power stability	0.701	11.946	8.370	4.24
Energy conversion efficiency	1.432	12.302	17.621	8.93
Communication distance attenuation rate	0.175	12.162	2.129	1.08
Packet loss rate	0.602	11.657	7.018	3.56
Data validation	0.694	12.104	8.396	4.25
Timeliness	2.851	12.706	36.221	18.35
Measurement accuracy	0.499	12.439	6.206	3.14
Measurement specifications	0.965	12.147	11.722	5.94

**Table 5:** Weight of the subjective and objective fusion.

Parameter Type	Parameters	Weight (%)
Energy harvester index X1	Open-circuit voltage deviation rate X11	5.68
	Short-circuit current deviation rate X12	8.81
	Equivalent resistance deviation rate X13	2.17
Energy management circuit index X2	Output power stabilityX14	6.69
	Output voltage stability X21	7.52
	Output power stability X22	21.39
Wireless communication index X3	Energy conversion efficiency X23	9.70
	Communication distance attenuation rate X31	0.86
	Packet loss rate X32	2.44
	Data packet efficiencyX33	4.12
Sensor unit index X4	Timeliness X34	11.09
	Measurement accuracy X41	14.09

**Table 6:** Specific parameters of sensors.

Evaluation Index	Parameter
Open-circuit voltage deviation rate	13.82%
Short-circuit current deviation rate	10.40%
Equivalent impedance deviation rate	3.13%
Output power stability	8.17%
Output voltage stability	5.57%
Circuit output power stability	1.59%
Energy conversion efficiency	77.75%
Communication distance attenuation rate	0.60%
Packet loss rate	1.7%
Data packet efficiency	98%
Timeliness	85.26%
Measurement accuracy	2 °C
Measurement accuracy	159 °C

**Table 7:** Standard values of the evaluation indexes.

Evaluation Index	V1	V2	V3	V4
X11 (%)	11.41	22.83	34.24	45.65
X12 (%)	17.76	35.52	53.28	71.04
X13 (%)	10.71	21.43	32.14	42.85
X14 (%)	18.25	36.5	54.75	73
X21 (%)	11.36	22.73	34.09	45.45
X22 (%)	5	10	15	20
X23 (%)	80.35	67.34	54.85	42.25
X31 (%)	10	30	40	60
X32 (%)	2.5	5	7.5	10
X33 (%)	98	96	94	92
X34 (%)	95	85.55	76.11	66.66
X41 (°C)	1	3	6.5	9
X42 (°C)	160	150	140	130

**Table 8:** Membership model of each index.

Type Layer	Factor Layer	Selected Membership Model
Energy harvester	Open-circuit voltage deviation rate	Model 1
	Short-circuit current deviation rate	Model 1
	Equivalent resistance deviation rate	Model 2
	Output power stability rate	Model 2
Energy management circuit	Output voltage stability	Model 2
	Output power stability	Model 2
	Energy conversion efficiency	Model 2
Wireless communication unit	Communication distance attenuation rate	Model 1
	Packet loss rate	Model 1
	Data packet efficiency	Model 2
	Timeliness	Model 2
Sensing unit	Measurement accuracy	Model 2
	Measurement range	Model 2

## Data Availability

The data used to support the findings of this study are included in the article.
